# Plasticizers: negative impacts on the thyroid hormone system

**DOI:** 10.1007/s11356-022-19594-0

**Published:** 2022-03-18

**Authors:** Ceyhun Bereketoglu, Ajay Pradhan

**Affiliations:** 1 Department of Bioengineering, Faculty of Engineering, Marmara University, 34722 Istanbul, Turkey; 2 Biology, The Life Science Center, School of Science and Technology, Örebro University, 701 82 Örebro, Sweden

**Keywords:** Thyroid, Thyroid hormones, Phthalates, Environmental pollutants, Metabolism

## Abstract

This review aims to understand the impacts of plasticizers on the thyroid system of animals and humans. The thyroid gland is one of the earliest endocrine glands that appear during embryogenesis. The thyroid gland synthesizes thyroid hormones (TH), triiodothyronine (T3), and thyroxine (T4) that are important in the regulation of body homeostasis. TH plays critical roles in regulating different physiological functions, including metabolism, cell growth, circadian rhythm, and nervous system development. Alteration in thyroid function can lead to different medical problems. In recent years, thyroid-related medical problems have increased and this could be due to rising environmental pollutants. Plasticizers are one such group of a pollutant that impacts thyroid function. Plasticizers are man-made chemicals used in a wide range of products, such as children’s toys, food packaging items, building materials, medical devices, cosmetics, and ink. The increased use of plasticizers has resulted in their detection in the environment, animals, and humans. Studies indicated that plasticizers could alter thyroid function in both animals and humans at different levels. Several studies demonstrated a positive and/or negative correlation between plasticizers and serum T4 and T3 levels. Plasticizers could also change the expression of various TH-related genes and proteins, including thyroid-stimulating hormone (TSH), thyrotropin-releasing hormone (TRH), and transporters. Histological analyses demonstrated thyroid follicular cell hypertrophy and hyperplasia in response to several plasticizers. In conclusion, plasticizers could disrupt TH homeostasis and the mechanisms of toxicity could be diverse.

## Introduction

Different types of compounds have been used as a plasticizer. In the 1850s, castor oil was used as a plasticizer, but in the 1870s, camphor became the popular plasticizer. However, camphor’s high volatility and unpleasant odor motivated the search for an alternative. In 1920, phthalate was discovered, and in 1933, the introduction of di-2-(ethylhexyl) phthalate (DEHP) opened the market for softer polyvinyl chloride materials (Graham 1973). At present, there are more than 100 different plasticizers produced worldwide, but only half of them are commercially important (Godwin 2017). Their common classifications are based on their chemical composition. The main types of plasticizers are phthalate esters, dibasic acid ester, epoxy plasticizers, glycol derivatives, benzoates, trimellitates, phosphate ester, and citrate (Bui et al. 2016).

Plasticizers are used in numerous products, such as children’s toys, household items, medical devices, clothing, food packaging materials, construction materials, and cosmetics (Kavlock et al. 2002). Plasticizers are added in plastic materials to increase their plasticity, flexibility, and durability, while in cosmetics to hold color and scent. They are also used as solvents in paints, glue, and insect repellent. Plasticizers are not covalently bound to the products; hence, they can easily leach out from the material into the environment (Bauer and Herrmann 1997). Due to this, plasticizers are accumulating in the ecosystems and detected in aquatic systems, drinking water, air dust, soils, and sewage treatment plants at substantial concentrations. Plasticizers are also found in food items, including milk products, meat, fish, and other high-fat-containing food (Barnabe et al. 2008; Bilal et al. 2019; Horn et al. 2004; Larsson et al. 2017). Hence, there are concerns over their use in everyday items.

Humans and animals are exposed to plasticizers through inhalation, oral ingestion, and skin contact. Plasticizers have negative effects on different physiological functions, including the immune system, metabolism, reproduction, brain function and cell differentiation (Barakat et al. 2017; Dobrzynska, 2016; Heudorf et al. 2007). The global market of plasticizers is expected to reach 10.5 billion tonnes worldwide by 2026 (Caresana, 2019). Hence, it is essential to determine their toxicities and monitor their environmental levels to avoid long-term impacts on humans and animals. Plasticizers are also known to influence the thyroid hormone (TH) system. The alteration of thyroid homeostasis by plasticizers could be through binding to TH receptors or carrier proteins, including transthyretin (TTR), and altering the transport protein, including sodium iodide symporter (NIS) (Boas et al. 2012; Zoeller, 2005). This review aims at understanding the impacts of plasticizers on the TH system in animals and humans.

## Commonly used plasticizers and their toxicity

### Di-(2-ethylhexyl) phthalate (DEHP)

DEHP is commonly used as a plasticizer in a wide range of plastic materials, such as medical devices, pharmaceuticals, cosmetics, personal care products, consumer products, building, and furniture materials (Kastner et al. 2012; Magdouli et al. 2013). DEHP is the most widely used phthalate, and as a result, it is abundant in the environment (Gimeno et al. 2014). The safe limit of DEHP concentration in drinking water has been determined as 6 and 8 µg/L by the US Environmental Protection Agency (U.S. EPA, 2012) and the World Health Organization (WHO, 2011), respectively. According to US EPA Chemical Data Reporting (CDR) database, the production volume of DEHP ranged between 45,359 and 113,398 tonnes in 2015 (U.S. EPA, 2020a).

Among the phthalates, DEHP contamination is highest in the aquatic environments with concentrations up to 97.8 µg/L in surface waters, 182 µg/L in sewage effluents, 154 mg/kg (dry weight) in sewage sludge, and 8.44 mg/kg in sediments (Fromme et al. 2002; Magdouli et al. 2013; Wang et al. 2008; Zeng et al. 2008). DEHP metabolite, mono-(2-ethylhexyl) phthalate (MEHP), has been reported to be up to 1.3 µg/L in river (Suzuki et al. 2001) and 57.2 ng/L and 0.84 ng/g dry weight in seawater and sediments, respectively (Blair et al. 2009). DEHP has also been detected in poultry, cooking oils, and cream-based dairy products with concentrations above 300 µg/kg (Serrano et al. 2014).

Several studies have demonstrated that DEHP is toxic and has potential carcinogenic properties, and due to this, it is listed as a category 1B chemical (Bui et al. 2016). In rats, DEHP negatively impacted reproduction, as it decreased sperm number and delayed vaginal opening (Andrade et al. 2006; Grande et al. 2006; Lee et al. 2009). DEHP is also known to increase lipid accumulation in different model systems by altering key genes in the fatty acid metabolism (Chen et al. 2016; Zhang et al. 2017).

### Di-butyl phthalate (DBP)

DBP is widely used in several products, including medical devices, cosmetics, paper coating, plastic piping, and food wrap. It is indicated that humans are exposed to DBP through water, air, and food items (Pan et al. 2006; Wei et al. 2011). DBP has been reported at concentrations ranging from 0.6 µg/L to 1472 mg/L in the environment (Fatoki and Noma, 2002; Fatoki and Ogunfowokan 1993; He et al. 2019; Paluselli et al. 2018; Tyler et al. 1998). The limit concentration of DBP in drinking water has been determined to be 3 µg/L (Sha et al. 2007). According to the European Food Safety Authority (EFSA), the tolerable daily intake of DBP has been suggested to be 10 µg/kg body weight (bw)/day, but it has been shown that the daily intake in China is 12.2 µg/kg bw/day (Guo et al. 2011).

Several studies have linked DBP with adverse health effects. A study demonstrated that exposure to DBP could induce histopathological changes in the spleen and cause oxidative stress leading to inflammation and apoptosis (Wang et al. 2020). DBP was shown to decrease body weight and increase maternal death, malformations in fetuses, and post-implantation loss during pregnancy in rats (Ema et al. 1993). Studies on zebrafish showed that DBP has teratogenic effects (Ortiz-Zarragoitia et al. 2006), alters plasma sex hormone levels (Chen et al. 2019), and results in abnormal heart rate and pericardial edema (Mu et al. 2020).

### Diethyl phthalate (DEP)

DEP is extensively used as a softener, coating material, and cosmetic additive in different products, such as hair sprays, perfumes, nail polish solvents, after-shave lotions, detergents, and shampoos (Kamrin and Mayor 1991; Kang et al. 2010). DEP is easily leached into the environment and can contaminate biological systems during DEP-containing product’s synthesis and use (Kang et al. 2010). In a survey performed in 1999–2000 on 2540 samples collected from participants of the National Health and Nutrition Examination Survey, DEP and its metabolite monoethyl phthalate (MEP) were detected in more than 75% of the urine samples (Silva et al. 2004). DEP has been detected at concentration of 0.6 µg/L in river water samples (Fatoki and Vernon 1990). The maximum detected levels of DEP were 632.2 mg/kg in household dust, 5481 ng/m^3^ in the indoor air of apartments, and 1263 ng/m^3^ in the indoor air of kindergartens (Fromme et al. 2004). The minimal risk level of DEP was determined to be 7 mg/kg bw/day in the USA (Duty et al. 2005). DEP was detected in human semen with a mean level of 0.47 μg/L (Zhang et al. 2006).

In vitro treatment of human high-density lipoproteins with DEP resulted in oxidation, aggregation, and degradation of lipoproteins and promoted foam cell formation (Kim et al. 2015). In the same study, exposure of zebrafish embryos to DEP showed embryo death, developmental speed decrease, and skeletal development impairment (Kim et al. 2015). Exposure to DEP was shown to alter liver and serum enzyme levels in young male Sprague–Dawley rats (Sonde et al. 2000), while other studies did not find any impact of DEP in rats (Foster et al. 1980) and mice (Lamb et al. 1987).

## Alternative plasticizers and their toxicity

The reported toxicity of DEHP and other phthalates has resulted in the search for alternatives with low toxicity. Henceforth, alternative plasticizers have been introduced without sufficient risk assessment, and their use is overtaking the traditional plasticizers (Bui et al. 2016). In many products, including phones and computers, the banned plasticizers are excluded, but it is not specified which alternative plasticizers are being used (Pecht et al. 2018). In the European market, the use of alternative plasticizers has increased steadily. Of the alternative plasticizers, diisononyl phthalate (DINP) and diisononyl cyclohexane-1,2-dicarboxylate (DINCH) are the most widely used worldwide.

### Diisononyl phthalate (DINP)

DINP is a high molecular weight alternative plasticizer with a backbone of 7–13 carbon atoms. This increases its permanency and durability (Revathy and Chitra 2018). DINP is used in different materials, such as household products, food wrapping, cosmetics, toys, paints, lubricants, and adhesives (Agency 2010). Since it is not chemically bound to polymers, DINP can enter the environment via leaching, migration, evaporation, and/or abrasion (Forner-Piquer et al. 2018). DINP has been detected in the floor and multi-surface dusts with 100% detection rates and maximum concentrations of 2100 and 15,500 µg/g dust, respectively (Ait Bamai et al. 2014). DINP metabolites have also been detected in human urine samples (Koch et al. 2013). As reported by the CDR database, the production volume of DINP was between 100 and 500 million pounds in the USA by 2015 (U.S. EPA, 2020b).

Several mammalian studies have found negative impacts of DINP on the reproductive system, including induction of nipple retention, alteration of sperm number and motility, decrease in testosterone level, and degeneration in testis (Boberg et al. 2011; Borch et al. 2004; Gray et al. 2000; Masutomi et al. 2003). Other studies have indicated that DINP can result in aggravated allergic dermatitis (Kang et al. 2016) and allergic asthma (Hwang et al. 2017) in mice. Studies on zebrafish demonstrated that exposure to DINP could decrease the number of fertilized eggs (Forner-Piquer et al. 2018) and lead to abnormal gonadal development and reproduction (Santangeli et al. 2017).

### Diisononyl cyclohexane-1,2-dicarboxylate (DINCH)

DINCH was introduced in 2002 to replace DEHP in food contact materials. DINCH received final approval from the European Union in 2006 (Bui et al. 2016). DINCH is widely used in polyvinyl products, including children’s toys and medical devices (EFSA, 2007). The annual production of DINCH is more than 10,000 tonnes in the European market (ECHA 2021). As the use of DINCH is increasing, its level in the environment is becoming prominent (Larsson et al. 2017). In urine samples from the German Specimen Bank, cyclohexane-1,2-dicarboxylic acid monoisononyl ester (MINCH) metabolites, OH-MINCH, cx-MINCH, and oxo-MINCH were detected at concentrations of 2.09, 0.86, and 1.81 μg/L, respectively (Schutze et al. 2014). Other studies have also detected different DINCH metabolites in human urine samples (Fromme et al. 2016; Gomez Ramos et al. 2016; Kasper-Sonnenberg et al. 2019; Minguez-Alarcon et al. 2016; Schwedler et al. 2020).

Like other alternative plasticizers, the toxicity of DINCH is not properly understood. Several studies have investigated DINCH toxicity; however, the data is contradictory (Campioli et al. 2015; Campioli et al. 2019; Campioli et al. 2017; David et al. 2015; EFSA, 2007; Engel et al. 2018; Nardelli et al. 2017; Vasconcelos et al. 2019). For instance, some studies have not observed any effect on behavior, organ weight, serum chemistry (David et al. 2015), and reproduction (EFSA, 2007). However, other studies showed that DINCH and/or its metabolites caused thyroid hyperplasia and renal toxicity (EFSA, 2007), changed the expression of genes involved in lipid metabolism (Campioli et al. 2015; Campioli et al. 2019), and altered Leydig cell function, and resulted in testicular atrophy in rats (Campioli et al. 2017).

## Thyroid hormone (TH) system

THs are produced by the thyroid gland, which sits on the neck region (Barrett et al. 2018). The name thyroid was derived from a Greek word “*thyreos*” for shield due to its shield-like appearance (Ellis 2011). Thyroid is the only endocrine gland that can produce and store the two THs, triiodothyronine (T3) and thyroxine (T4). T4 is the major TH secreted by the thyroid gland, whereas T3 is the main biologically active form (Dezonne et al. 2015; Izumi and Larsen 1977). From the thyroid gland, the THs are transported to different tissues. The thyroid is the main site for T4 and T3 synthesis; however, T3 synthesis also takes place in the peripheral tissues. In the target tissues, iodothyronine deiodinase (DIO) enzymes play an essential role in regulating the levels of THs. There are three types of deiodinase enzymes (DIO1, DIO2, and DIO3) that are involved in TH synthesis and regulation (Gereben et al. 2015). The deiodinases are unique enzymes as they contain selenocysteine group. DIO2 catalyzes the conversion of T4 to T3 following deiodination of the outer ring of T4 (Bianco et al. 2002; Peeters and Visser 2000). DIO1 can also generate T3 from T4 but has a lower affinity and high Km for T4 than DIO2. DIO1 and DIO3 can metabolize both T3 and T4, thereby diminishing TH signaling (Gereben et al. 2015). DIO1 and DIO3 can convert T4 to inactive T3 (rT3) by deiodination of the inner ring. DIO1 and DIO3 can further metabolize T3 (by deiodination of the inner ring) and rT3 (by deiodination of the outer ring) (Bianco et al. 2002; Peeters and Visser 2000). The tissue distribution of DIO1 and DIO3 is different, with DIO1 predominantly expressed in the liver, kidney, and thyroid, while DIO3 is largely expressed in the brain. Hence, DIO3 could be important in regulating T3 levels in the brain. On the other hand, DIO2 is expressed in the brain, esophagus, and thyroid (Peeters and Visser 2000). Environmental pollutants, including polybrominated flame retardants (PBDEs) are known to inhibit DIO2 enzymatic activity and reduce T3 synthesis in H4 glioma cells (Roberts et al. 2015). In zebrafish, a pesticide azocyclotin was shown to reduce T3 levels by altering *Dio2* level (Jiao et al. 2019).

**Fig. 1:**
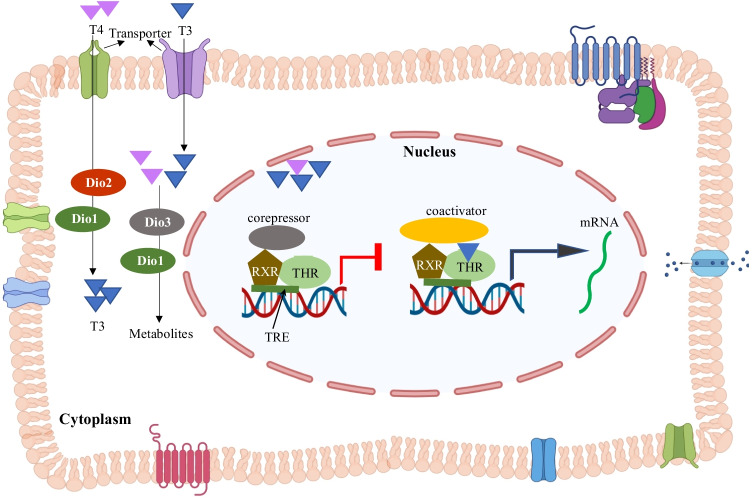
**Thyroid Hormone Signaling and Regulation**. Thyroid hormones (THs), triiodothyronine (T3) and thyroxine (T4,) enter the target cell through transporters present on the cell membrane. Once inside the cell, T4 gets converted to T3, primarily by type II iodothyronine deiodinase (DIO2) enzyme and to a low extent by type I iodothyronine deiodinase (DIO1). T4 and T3 are converted to inactive metabolites by DIO1 and type III iodothyronine deiodinase (DIO3). TH can bind to thyroid hormone receptors (THRs) in the nucleus and carry out gene expression. The THR makes a complex with retinoid X receptor (RXR) and binds to the thyroid response element (TRE) on the upstream region of the gene transcription start site. In a resting or uninduced state, the receptor complex binds to corepressor, which blocks the gene transcription. The binding of TH to THR leads to a conformational change of THR, which releases corepressor, and coactivators bind to start the transcription of TH response genes. The figure was generated using Biorender software

The synthesized TH exerts its action by interacting with thyroid hormone receptors (THRs; THRα and THRβ) located in the nucleus. Following activation of THRα or THRβ by TH, corepressors dissociate from THR due to conformational changes, and coactivators are recruited, and this complex drives the transcription of thyroid-regulated genes (Fig. 1) (Brent 2012). TH plays a crucial role in regulating different aspects of animal physiology, including energy homeostasis, circadian rhythm, cellular growth, and development (Baksi and Pradhan 2021; Brent 2012; Ikegami et al. 2019; Mullur et al. 2014). TH also influences brain development, as it is involved in neurogenesis, neuronal migration, neuronal and glial differentiation, myelination, and synaptogenesis (Lima et al. 1998; Manzano et al. 2007; Martinez-Galan et al. 1997; Schoonover et al. 2004). Alteration in TH, either low (hypothyroidism) or high (hyperthyroidism), can cause problems related to bone (osteoporosis), brain (cognition, visual attention, visual processing, motor skills, language, and memory skills), fatigue, and temperature intolerance (Williams 2008). Neuro-psychiatric disorders such as schizophrenia, bipolar disorder, anxiety, and depression are also associated with abnormal TH levels (Nandi-Munshi and Taplin 2015; Noda 2018).

Thyroid-related problems, including brain functions, are on the rise, and this could be attributed to the increasing level of environmental pollutants (Mughal et al. 2018). However, there is limited information about the effects of plasticizers on thyroid disruption and the molecular mechanisms of toxicity. Filling this knowledge gap will help understand the impacts of pollutants on physiological functions and screen new emerging chemicals for their disrupting properties. The health effects associated with phthalates and/or their metabolites on model organisms are summarized in Table 1.

**Table 1:** The health effects associated with phthalates/metabolites on model organisms. ↑ refers to an increase, while ↓ indicates a decrease

Phthalates/metabolites	Model organism	Exposure concentration	Sample size	Effects	Reference
DEHP	Zebrafish larvae Rats Wistar rats Rats Rat thyroid follicular cells Juvenile rats Juvenile Wistar rats Wistar rats	400 µg/L Oral gavage, 250, 500 and 750 mg/kg/day 150, 300, and 600 mg/kg 10000 ppm 9, 21, and 48 mg/kg bw/day 5, 50, and 500 mg/kg/day Oral gavage of 150, 300, and 600 mg/kg/day	90–600 larvae 24 males 40 males 20 males 5 males/5 females 40 males/40 females 128 females	T4↑, T3↑, thyroid related genes↑↓ TSHr↓, TRHr↑ Alteration in thyroid follicular cell hypertrophy and hyperplasia TT3, TT4, FT3 and FT4↓, Serum level of thyroid peroxidase↓ NIS↓ mRNA and protein level of TTR↓ T4 and FT3 levels↓ Alteration in TH level Alteration in expression of genes and proteins involved in TSH/TSHr signaling pathways Follicular cells↓ Follicular cells with a columnar appearance↑ Iodine uptake↑ Follicle areas, colloid areas, number of cells in follicular epithelium, and epithelium areas ↑(in female) TSH gene expression↓(in female) bw on hypothalamic-pituitary-thyroid axis↑ Protein levels of TRH in the hypothalamus↓ Protein and mRNA levels of thyrotropin-releasing hormone receptor in the pituitary↑ mRNA level of thyroid-stimulating hormone receptor↓ Alteration in the expression of mRNA and protein levels of TRHr, TSHβ, TSHr, TPO, TTF-1, TG	Jia et al. 2016 Liu et al. 2015 Wu et al. 2021 Howarth et al. 2001 Wenzel et al. 2005 Tassinari et al. 2021 Sun et al. 2018 Dong et al. 2017
MEHP	Zebrafish larvae	200 µg/L	30–1200 larvae	T4↓, T3↑, HPT axis genes↑↓	Zhai et al. 2014
DBP	*Xenopus laevis* tadpoles Rats Wistar rats	2, 10, or 15 mg/L 50 mg/kg 50 mg/kg	160 tadpoles 30 males 80 females	*THRβ*, *RXRγ*, *TSHα*, and *TSHβ*↑↓ Serum T3 and T4 levels↓ T3↓	Shen et al. 2011 Baralic et al. 2020 Y Wu et al. 2017a, b
MBP	*Xenopus laevis* tadpoles	2, 10, or 15 mg/L	160 tadpoles	*THRβ*, *RXRγ*, *TSHα*, and *TSHβ*↑↓	Shen et al. 2011
DIDP	Rat thyroid follicular cells			Iodine uptake↑	Wenzel et al. 2005
DOP	Rat thyroid follicular cells			Iodine uptake↑	Wenzel et al. 2005
DINP	Rat thyroid follicular cells			Iodine uptake↑	Wenzel et al. 2005
DnHP	Rats	10000 ppm	20 males	Follicular cells↓ Follicular cells with a columnar appearance↑	Howarth et al. 2001

## The impacts of plasticizers on thyroid system

### Impacts on aquatic animals

Environmental pollutants, including plasticizers, can end up in the water bodies, and aquatic animals could be at greater risk. Hence, it is important to understand the toxicity of pollutants in different aquatic model systems. Many studies highlighting plasticizer’s toxicity on the TH system have been published. Exposure to the DEHP metabolite, MEHP (200 µg/L), reduced T4 level in zebrafish larvae, while the level of T3 was increased. Genes involved in hypothalamus-pituitary-thyroid (HPT) axis, *nis*, *pared box 8* (*pax8)*, *NK2 homeobox 1 (nkx2.1), thyroglobulin (tg)*, *ttr*, *dio1*, *dio2*, and *UDP glucuronosyltransferase 1 family polypeptide a3 isoform 1 (ugt1ab)* were affected at doses of 8–200 µg/L MEHP (Zhai et al. 2014). In another study using a similar experimental setup, Jia et al. (2016) showed that exposure of zebrafish larvae to 400 µg/L of DEHP can increase T4 and T3 levels (Jia et al. 2016). In the same study, DEHP altered the expression of several thyroid-related genes such as *thyroid-stimulating hormone beta (tshβ), corticotropin-releasing hormone (crh)*, *nkx2.1*, *tg,* and *dio2* in a dose-dependent manner (Jia et al. 2016). MEHP is known to be more toxic than DEHP (Pollack et al. 1985); hence, the higher toxicity of MEHP on the TH system is not surprising.

In an in vitro and in vivo study, Sugiyama et al. (2005) analyzed the effects of phthalates on the thyroid system. Using the luciferase assay, it was found that dicyclohexyl phthalate (DCHP), benzyl butyl phthalate (BBP), and DBP can inhibit T3 activity with IC50 of 11 ± 3 µM, 40 ± 6 µM and 39 ± 1 µM, respectively. In in vivo experiment, *Xenopus laevis* tadpoles immersed in buffer with 2-nM T3 alone or in combination with phthalates showed that co-treatment with BBP can significantly downregulate T3 dependent induction of *THR beta* (*THRβ*) gene (Sugiyama et al. 2005). In a similar study, African clawed frog tadpoles exposed to 2, 10, or 15 mg/L DBP or mono butyl phthalate (MBP) for 21 days showed that the two phthalates at all the concentrations can alter the expression of several thyroid-related genes, including *THRβ*, *retinoid X receptor gamma (RXRγ)*, *TSHα*, and *TSHβ*. An in vitro analysis further showed that DBP and MBP could induce the interaction between silencing mediator for retinoid and thyroid hormone receptors (SMRT) and THR in a dose-dependent manner. The authors highlighted that DBP and MBP have a potential to disrupt thyroid activity (Shen et al. 2011).

### Impacts on rodents

Several studies have been conducted in rodent models to analyze the effects of plasticizers on the TH system. DEHP exposure on rats (oral gavage, 250, 500, and 750 mg/kg/day) resulted in alteration of genes involved in TH signaling (Liu et al. 2015). *TSH receptor (TSHr)* expression was significantly downregulated in response to 500 and 750 mg/kg/day DEHP, while *thyrotropin-releasing hormone receptor (TRHr)* was significantly upregulated in a dose-dependent manner (Liu et al. 2015). Histological analysis indicated that DEHP could alter thyroid follicular cell hypertrophy and hyperplasia in 500 and 750 mg/kg/day doses. Total T4 (TT4) and free T4 (FT4) were reduced by 500 and 750 mg/kg/day doses; however, no effect was observed on TSH level. Total T3 (TT3) and free T3 (FT3) were also reduced by 750 mg/kg/day dose, while only TT3 was reduced by 500 mg/kg/day dose. Serum level of thyroid peroxidase (TPO) was reduced by both 500 and 750 mg/kg/day doses, while NIS was reduced by only 750 mg/kg/day dose (Liu et al. 2015). mRNA and protein levels of TTR were downregulated by all the doses of DEHP. Based on the findings, the authors argued that the DEHP-mediated alteration of TH could be due to changes in biosynthesis, transformation, transport, and metabolism of TH (Liu et al. 2015).

Other studies have also investigated whether there was an association between phthalates and thyroid profiles. Baralic et al. (2020) showed that rats orally dosed for 27 days with DEHP (50 mg/kg/day), DBP (50 mg/kg), BPA (25 mg/kg), and a mixture of these three chemicals can alter thyroid profile. Serum content of T3 and T4 showed a negative association with DBP and BPA (Baralic et al. 2020). Supporting this, Wu et al. (2017a, b) found that oral exposure to DBP (50 mg/kg/day) for 5 weeks can decrease T3 levels in female Wistar rats (Wu et al. 2017b). In a recent study, Wu et al. (2021) analyzed the effects of DEHP (150, 300, and 600 mg/kg) on the HPT axis using a total of 40 Wistar rats (2 weeks old) upon intragastric administration for 90 days. Although the levels of THs, FT3, and FT4 did not change in 150 mg/kg/day exposure group, the levels of T4 and FT3 were significantly decreased in response to 300 mg/kg/day exposure group. The levels of T3 and TSH in the 600 mg/kg/day exposure group were also significantly altered. DEHP exposure also led to histological changes in the thyroid gland. Besides, DEHP resulted in altered expression of genes and proteins involved in TSH/TSHr signaling pathways. Altogether, the authors concluded that DEHP could disrupt TH homeostasis through different mechanisms (Wu et al. 2021). In a study, chronic exposure to BPA (200 mg/kg bw/day for 35 days) resulted in significantly decreased serum levels of T3 and T4 accompanied by an increased serum TSH level in rats. The authors supported their findings through histological analysis indicating severe pathological changes in the thyroid tissue of BPA-treated rats (Mohammed et al. 2020).

Analysis of six plasticizers using rat thyroid follicular cells, Wenzel et al. (2005) showed that DEHP, di-isodecyl phthalate (DIDP), di-octyl phthalate (DOP), and DINP can enhance iodide uptake by regulating NIS. Other plasticizers, including BBP and DBP, showed no effect on iodide uptake (Wenzel et al. 2005). Analysis of DEHP and di-n-hexyl phthalate (DnHP) on male rats showed alteration in thyroid tissue. Exposure to phthalates, DEHP, and DnHP alone or in combination (10 000 ppm concentration for both) resulted in reduction of follicular cells and increase in the number of follicular cells with a columnar appearance, a sign of hyperactivity (Howarth et al. 2001). Interestingly, the effect of DnHP was stronger in thyroid tissue but did not alter other parameters, such as liver weight, induction of fatty acid oxidation, and CYP4A1 like DEHP (Howarth et al. 2001).

The physiology in organisms, including the thyroid system and TH levels, differ in females and males. This could result in sex-specific effects of environmental pollutants, and revealing this may help better understand the toxicity of pollutants. Several studies have demonstrated that phthalates have sex-specific effects on TH activities. Tassinari et al. (2021) orally exposed juvenile rats to various concentrations of DEHP (0, 9, 21, and 48 mg/kg bw/day) for 28 days and analyzed different parameters. Histomorphometric analysis indicated that DEHP demonstrated no significant change in male rats, while thyroid follicle areas, colloid areas, and number of cells in follicular epithelium significantly increased in female rats in response to 9 and 48 mg/kg bw/day groups. Besides, epithelium areas significantly increased in female rats in 9 mg/kg bw/day group. *TSH* gene expression was significantly downregulated in females in 48 mg/kg bw/day group. Altogether, it was suggested that DEHP could alter thyroid homeostasis in immature rats (Tassinari et al. 2021). TH signaling shows sex-specific differences (Baksi and Pradhan 2021), and plasticizer-mediated sex-specific toxicity (Tassinari et al. 2021) could be an important indication that both males and females are needed to assess the toxicity of plasticizers on the TH system.

**Fig. 2:**
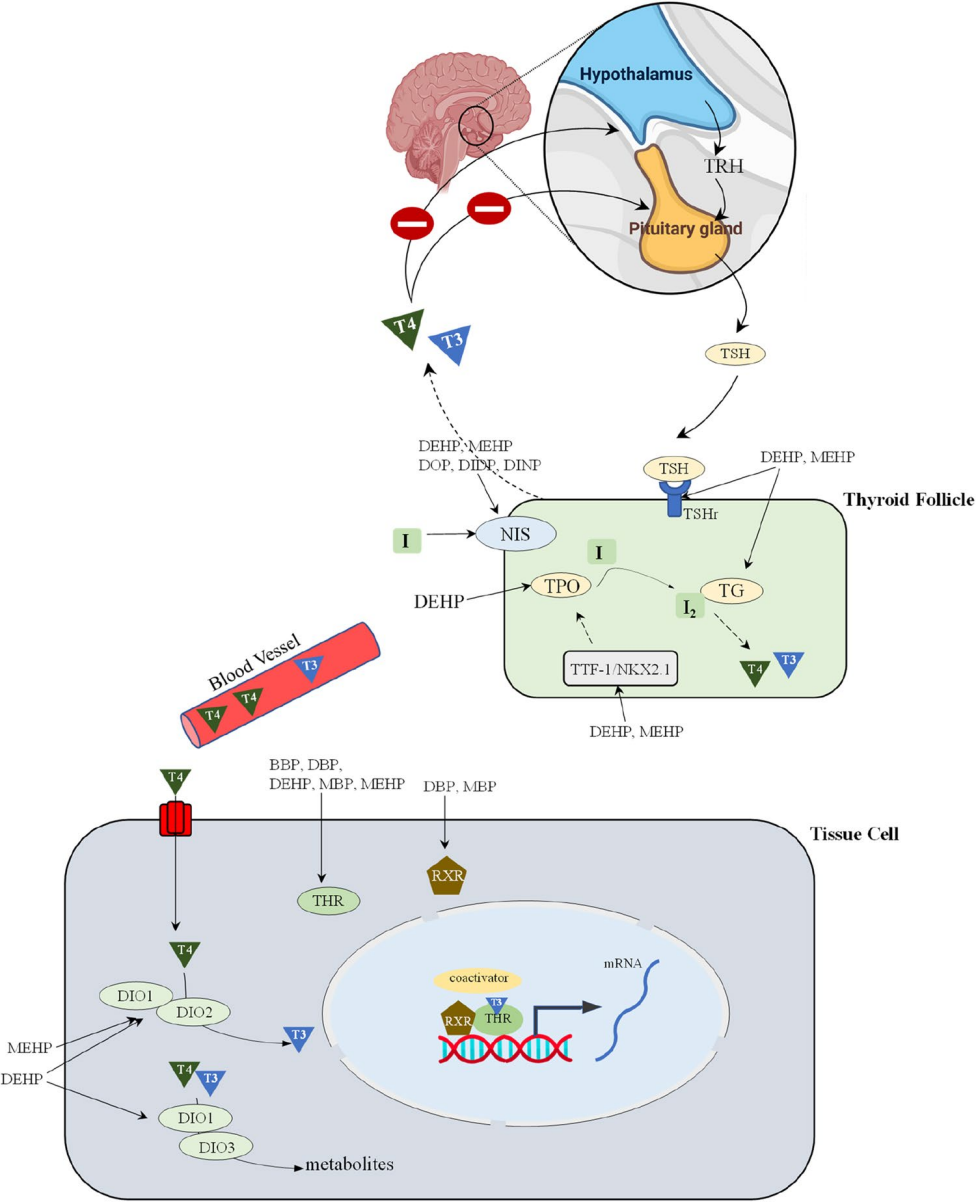
**Schematic representation of thyroid signaling and potential mechanisms of toxicity of different plasticizers**. The hypothalamus and pituitary regulate the thyroid gland. Hypothalamus secretes thyrotropin releasing hormone (TRH) that acts on pituitary, resulting in the release of thyroid stimulating hormone (TSH). TSH acts on the thyroid to induce the production of THs (thyroxine: T4 and triiodothyronine: T3), which in turn act on the hypothalamus and pituitary for negative feedback regulation. T4 and T3 produced in the thyroid gland reach other tissues through the circulatory system. TH can bind to thyroid hormone receptor (THRs) in the nucleus and regulate gene expression. Plasticizers are known to impact different steps of TH signaling. For instance, they can bind to THRs, retinoid X receptor (RXR), thyroid stimulating hormone receptor (TSHr), thyroglobulin (TG). Plasticizers can also influence TH signaling by regulating enzymes, including DIO1, DIO2 and DIO3 involved in TH synthesis and metabolism. Although plasticizers have been shown to impact transcript levels of deiodinase enzymes, it is not known if they can alter TH synthesis by directly affecting deiodinase enzyme activity at the protein level

Hypothalamus and pituitary are important for regulating thyroid function, and the released TH from the thyroid can have negative feedback on them (Fig. 2). This loop, the hypothalamic-pituitary-thyroid (HPT) axis, is critical for maintaining different physiological functions. Plasticizers are suggested to disrupt the HPT axis, and few studies have highlighted this. In a study, juvenile Wistar rats were exposed to 0, 5, 50, and 500 mg/kg/day DEHP for 28 days by oral gavage to analyze the effect of DEHP on HPT axis. It was demonstrated that higher concentrations of DEHP can decrease protein levels of TRH in the hypothalamus, increase protein and mRNA levels of TRHr in the pituitary, and decrease mRNA level of TSHr in the thyroid. The authors concluded that DEHP could interfere with the thyroid homeostasis in juvenile rats (Sun et al. 2018). Dong et al. (2017) investigated the effect of various concentrations of DEHP (oral gavage of 150, 300, and 600 mg/kg/day for 3 and 6 months) on THs in Wistar rats (128 rats) at the transcript and protein levels. The authors observed that DEHP can alter mRNA and protein levels of TRHr, TSHβ, TSHr, TPO, thyroid transcription factor 1 (TTF-1), and TG. The authors suggested that exposure to DEHP could affect TH levels by altering HPT axis of the body through TSH/TSHr signaling (Dong et al. 2017).

### Impacts on humans

Humans can be exposed to plasticizers through multiple routes, including oral, inhalation, and dermal. It is likely that humans are getting exposed to plasticizers from the day-to-day items surrounding them. In 2011, there was a food scandal in Taiwan where the two plasticizers DEHP and DINP were purposely added by the companies in their items. Of the 965 contaminated products, 206 were exported to 22 countries (Yang et al. 2013). This indicates that humans may be exposed to phthalates continuously through various routes and sources. Phthalates are rapidly degraded into their metabolites and excreted in urine and serum upon exposure (Hauser and Calafat 2005). Phthalates may change physiological conditions in humans directly or through their metabolites. Several epidemiological studies have analyzed the association of phthalate exposure with the human thyroid system and hormone levels (Table 2). Wu et al. (2013) analyzed 60 children of 4–6 years old upon exposure to DEHP (29 high dose, 23 low dose, and 8 non-exposed) and found that the serum TSH levels were significantly lower in the high- and low-dose groups. However, T3, T4, and FT4 levels were not different from the non-exposed group. Interestingly, 6-month follow-up study showed no change in TSH, but T3 level was significantly altered in the high dose group (*n* = 13). In this study, it was indicated that FT3 is more relevant for biological action than T3 (Wu et al. 2013). Later, the same group performed a study with 250 participants that were exposed to plasticizers. However, no significant association was found with the serum TSH, T4, T3, and FT4 levels (Tsai et al. 2016). Similar results were obtained from another study that was performed on 329 teenagers (12–19 years) and 1346 adults (above 19 years) (Meeker and Ferguson, 2011). The authors found that the urinary DEHP metabolites levels in adults were inversely associated with T3, T4, FT4, and TG, whereas a positive association was observed with TSH. Interestingly, DEHP metabolite showed a positive association with T3 and TSH in teenagers (Meeker and Ferguson, 2011). Since thyroid-related problems are known to show sex-specific and age-dependent prevalence (Cho et al. 2018; Gietka-Czernel 2017), this study further encourages the analysis of pollutant’s impacts on different age groups and sex. Meeker et al. (2007) analyzed urine samples from 408 men (18–55 years old) and measured phthalate metabolites, T4, T3, and TSH levels. MEP was detected in all the 408 men, and it showed negative and positive association with TT3 and TSH, respectively. The authors noted a weak inverse association between MEHP and FT3 (Meeker et al. 2007). As the authors also pointed out, the drawback of this study was that TT4 and FT3 levels were not measured.

**Table 2:** Human studies and their outcomes associated with phthalates/metabolites exposure/detection

Phthalates/metabolites	Timing of exposure/detection	Sample size	Measurement/exposure	Effects	Reference
DEHP DEHP DEHP MEHP MEHHP MEOHP MBzP MEP, MBP, MBzP, MEHP, MEHHP, MEOHP, MECPP, MOP, MiNP, MHiNP, MOiNP, MCiOP MEHP MBzP MEHHP MOP MMP MEP DEHP BBzP DBP MEHHP MBzP MnBP MEP MBzP MCCP DEHP Phthalate metabolites MEHP MEHHP MBP MEHHP MEOHP MBzP MEHP MEOHP MEHHP MiBP MnBP mPAEs DEHP MMP MEHHP MEHP MBP DEHP MEHP MEHHP 5-oxo-MEHP	Children (4–6 years old) Teenagers (12–19 years) Adults (above 19 years) Adults (≥ 18 years old) and minors (< 18 years old) Children (4–9 years old) Men (18–55 years old) Adults Adults Male Female Pregnant women (16–26 weeks of gestation) Pregnant women (16–26 weeks of gestation) Pregnant women Pregnant women Pregnant women Mother–child pairs Maternal-infant pairs Children Children (5–7 years old) Adults Adults Meta-analysis	60 329 teenagers 1346 adults 279 adults 79 minors 845 408 317 6003 6003 468 106 439 2521 76 181 pairs 148 pairs 189 216 55 44 thyroid cancer 138 benign nodule patients 144 healthy adults 116 13 articles	High/low exposure Urine/serum Urine/serum Urine Urine Urine Urine Urine/serum Urine/serum Urine/serum Urine/serum Urine/plasma Urine Maternal and cord sera Urine/serum Urine Urine/blood Urine Urine Urine Urine Urine	Lower TSH levels T3 and TSH (positive correlation in teenagers) T3, T4, FT4, and TG (negative correlation in adults), TSH (positive correlation in adults) T4 (negative correlation in adults) T4 (positive correlation in adults) T4 (negative correlation in adults) T4 (positive correlation in adults) T4 (positive correlation in minors) Detection of metabolites in all urine samples FT3 and TT3 (negative correlation in girls) T3 (negative correlation), TSH (positive correlation) T3, T3/T4 ratio (positive correlation) T3, T4 (negative correlation) TSH (positive correlation) T4 (negative correlation) T3, TSH (negative correlation) T4, TSH (negative correlation) T3, T4 (negative correlation) Thyrotropin (negative correlation) Free and total thyroid hormones (positive correlation) Maternal TT4 (negative correlation with both metabolites) Maternal FT4 (negative correlation with MEHP) Maternal TSH (positive correlation with MEHHP) No association between thyroid hormone and cord serum T4, FT4 (negative correlation) T4 (negative correlation in mothers) T3 (positive correlation in boys) TSH (negative correlation in cord blood) FT4 (positive correlation in girls) FT3 (positive correlation in boys) FT3, FT4 (positive correlation) Correlation between DEHP in serum and thyroid cancer malignancy Positive correlation with thyroid cancer Negative correlation with thyroid cancer Positive correlation with papillary thyroid cancer TT4 (negative correlation) Thyrotropin (positive correlation)	Wu et al. 2013 Meeker and Ferguson 2011 Huang et al. 2017b Boas et al. 2010 Meeker et al. 2007 Wang et al. 2018 Park et al. 2017 Park et al. 2017 Romano et al. 2018 Johns et al. 2015 Johns et al. 2016 Yao et al. 2016 Huang et al. 2007 Huang et al. 2017a Kuo et al. 2015 Weng et al. 2017 Weng et al. 2017 Wu et al. 2017a Marotta et al. 2019 Liu et al. 2020 Liu et al. 2020 Miao et al. 2020 Kim et al. 2019 Kim et al. 2019

Huang et al. (2017b) performed a study with 279 Taiwanese adults (≥ 18 years old) and 79 minors (< 18 years old) and investigated the association between phthalate exposure and thyroid function in 2013. The authors showed that the levels of urinary mono-(2-ethyl-5-hydroxyhexyl) phthalate (MEHHP) and the total of urinary DEHP metabolites were inversely correlated with T4 levels in adults, while FT4 levels were inversely correlated with urinary MEHP and mono-(2-ethyl-5-oxohexyl) phthalate (MEOHP) levels, and positively correlated with urinary MEP. Meanwhile, FT4 was positively correlated with urinary mono benzyl phthalate (MBzP) levels in minors (Huang et al. 2017b). This shows that phthalate exposure could affect thyroid function differently in adult and young populations. This differential regulation could be due to differences in metabolic activity.

Boas et al. (2010) analyzed the concentration of phthalate metabolites in urine samples of 845 Danish children aged 4–9 years to determine whether these metabolites are associated with thyroid functions. The authors detected all the 12 phthalate metabolites in all urine samples with the highest concentration of MBP. They also determined an inverse correlation between metabolites and serum levels of FT3 and TT3 in girls (Boas et al. 2010). This further suggests that there could be gender-specific effects of phthalate metabolites on TH system. It has been indicated that women show 5–20 times higher susceptibility to thyroid-related problems (Gietka-Czernel 2017). The underlying mechanisms are not clear and the differential action of pollutants could also be a contributing factor. Wu et al. (2017a) investigated the association between urinary concentrations of eight mono-phthalate metabolites (mPAEs) and thyroid function in China with 216 children aged 5–7 years. The authors found that mPAEs concentrations in children from urban areas were higher than those from rural areas. Most of the mPAEs positively correlated with FT3 and FT4 suggesting that phthalate exposure in children could affect TH (Wu et al. 2017a). In another study with 317 participants, including 165 workers engaged in waste plastic recycling and 152 farmers, it was shown that urinary levels of phthalates, particularly MBzP, MEHHP, and monooctyl phthalate (MOP) were significantly higher in workers compared to the controls (Wang et al. 2018). In contrast to a previous study (Boas et al. 2010), the phthalate metabolites were positively associated with T3 or T3/T4 ratio in all the participants. A non-monotonic dose–response relation between urinary phthalate metabolites and serum levels of TH, including mono-methyl phthalate (MMP) and T3 or T3/T4 ratio, and MEP and T3/T4 ratio was also demonstrated (Wang et al. 2018).

To show the association between phthalates and thyroid function, Park et al. (2017) measured the levels of several phthalate (DEHP, BBP, and DBP) metabolites in urine samples, and T3, T4, and TSH in serum samples. In this study, a large sample size of the Korean adult population involving 6003 participants during 2012–2014 was enrolled. The level of phthalate metabolites was associated with decreased T3 and T4 in urine samples, and increased TSH levels in serum samples. The authors showed that the level of MEHHP metabolite in urine inversely correlated with T4 in males, while MBzP and mono-n-butyl phthalate (MnBP) metabolites inversely correlated with T3 and TSH in females. Altogether, the authors suggested that exposure to phthalates have a potential to alter TH balance in adults (Park et al. 2017).

Exposure to phthalates during pregnancy may alter TH system, resulting in adverse effects on pregnancy, birth outcomes, and development of children (Villanger et al. 2020). The maternal urinary phthalate metabolites and TH levels have also been analyzed during pregnancy in different studies. Romano et al. (2018) measured the concentration of nine phthalate metabolites in urine samples from women at around 16–26 weeks of gestation, while they measured TSH, FT4, and TT4 and T3 in 202 maternal serums at 16 weeks of gestation and in 276 cord serums at delivery. A tenfold increase in maternal urinary MEP metabolite at 16 weeks of gestation was associated with decreased in maternal TT4. On the other hand, a tenfold increase in maternal urinary MBzP metabolite at both 16 and 26 weeks of gestation correlated with decreased cord serum TSH. This indicated that phthalate indexes in mothers and newborns negatively correlate with maternal serum TT4 and cord serum TSH, respectively (Romano et al. 2018). In a similar study, urine and serum samples were collected at 16 and 26 weeks of gestation in Northern Puerto Rico. Significant negative correlations were observed between mono-3-carboxypropyl phthalate (MCPP) and FT3 at 16 and 26 weeks of gestation, as well as DEHP and FT4 at 26 weeks of gestation. It was suggested that phthalate urinary metabolites could change maternal serum thyroid levels in a time-dependent exposure during gestation (Johns et al. 2015). Johns et al. (2016) further performed repeated measures of urinary phthalate metabolites and plasma TH levels in 439 pregnant women (116 cases and 323 controls) and demonstrated that phthalate metabolites negatively correlated with TSH and positively correlated with free and total TH. It was indicated that the time of exposure during gestation could affect the level and direction of these correlations (Johns et al. 2016). Derakhshan et al. (2021) analyzed the association of thyroid function with phthalate exposure in 1996 pregnant women. The authors found that higher DEHP and DINP metabolites were correlated with a lower FT4 and TT4, respectively. They also showed that higher concentrations of DBP and BBP metabolites were associated with lower T4/T3, higher FT4/TT4, and higher FT3/TT3. Higher DINCH metabolites were also found to correlate with higher TT3 (Derakhshan et al. 2021).

Yao et al. (2016) showed a link between phthalate exposure during the first trimester and TH in pregnant women and their newborns. In this study, the level of TH was measured in maternal and cord sera, while the level of seven phthalates was determined in urea. It was demonstrated that increase in MEHP and MEHHP associated with a decrease in maternal TT4, while the same metabolites were negatively and positively associated with maternal FT4 and maternal TSH, respectively. The authors did not observe any association between phthalate metabolites and TH in cord serum. Taken together, the authors suggested that exposure to phthalates during the first trimester could affect maternal TH levels (Yao et al. 2016). Huang et al. (2007) investigated 76 urine and serum samples from Taiwanese pregnant women in the second trimester to analyze whether there is an association between phthalate exposure and TH. The authors found that MBP, MEP, and MEHP were the predominant metabolites with median levels of 81.8, 27.7, and 20.6 ng/ml, respectively. They also determined a significant negative association between T4, FT4, and urinary MBP. Although DBP altered the thyroid function, the mechanism of action remains unclear (Huang et al. 2007).

The association between phthalates and mother–child pairs and children has also been investigated. Huang et al. (2017a) performed a meta-analysis with 181 mother–child pairs in central Taiwan and measured T3, T4, FT4, and TSH in children, as well as phthalate metabolites in urine samples. The authors showed that maternal DEHP metabolites, MEHHP and MEOHP, were negatively associated with T3 and T4 levels in boys. On the other hand, in girls, FT4 levels were negatively correlated with maternal urinary MEP, maternal urinary MBzP, and children’s urinary MEHP. The authors suggested that exposure to phthalates in early life could suppress TH levels in young children (Huang et al. 2017a). Kuo et al. (2015) performed a study on 148 Taiwanese maternal and infant pairs by collecting urine and blood samples in the third trimester of pregnant women and their cord blood samples at delivery. Among the detected nine phthalate metabolites, the urinary MBzP was found to be inversely associated with serum TSH in cord blood, suggesting that the parental compound of MBzP, BBP, could alter TSH function in newborns (Kuo et al. 2015). Weng et al. (2017) analyzed the effects of phthalates on thyroid function in 189 Taiwanese children based on gender and found a positive association between T4 and DEHP metabolites, MEHP, MEOHP, and MEHHP in girls, while in boys, there was a positive correlation between FT3 and DBP metabolites, mono-i-butyl phthalate (MiBP), and MnBP. It was also demonstrated that higher concentrations of DEHP have higher impacts on FT3 in boys. The authors suggested that phthalate metabolites could cause gender-specific effects on thyroid function (Weng et al. 2017).

Thyroid cancer is the most diagnosed cancer type (Miao et al. 2020) and several studies have suggested that the endocrine-disrupting chemicals may imbalance the TH system, leading to adverse outcomes, including cancer (Alsen et al. 2021; Marotta et al. 2020; Miao et al. 2020). Since several phthalates have been shown to have endocrine disruptive effects (Marotta et al. 2020; Villanger et al. 2020), it is critical to reveal their potential contributions to the etiology of thyroid disorders and cancers. Many studies have investigated the association between phthalates and thyroid cancer. Marotta et al. (2019) analyzed the association between DEHP and thyroid cancer development on 55 patients, 27 with a diagnosis of benign thyroid nodules, and 28 suffering from differentiated thyroid cancer. The authors observed a correlation between serum levels of DEHP and malignancy in dose-independent manner. However, this correlation was not mediated by higher TSH levels (Marotta et al. 2019). The authors indicated that the existence of DEHP in serum could result in a more than 14 times higher risk of developing differentiated thyroid cancer (Marotta et al. 2019). In another study, the role of urinary phthalate metabolites on the risk of thyroid cancer was investigated using sex-matched 44 thyroid cancer, 138 benign nodule patients, and 144 healthy adults from Wuhan, China (Liu et al. 2020). The authors demonstrated a positive correlation between three phthalate metabolites, MMP, MEHHP, and MEHP, and thyroid cancer. Meanwhile, urinary MBP and MBzP were found to be negatively correlated with thyroid cancer. The authors also indicated a gender-specific relation between phthalate metabolites and thyroid cancer, as MEHP significantly correlated in females but not in males (Liu et al. 2020). Miao et al. (2020) analyzed the link between urinary phthalate metabolites and papillary thyroid cancer and a positive correlation between DEHP metabolites and papillary thyroid cancer was observed. Although the results require confirmation, the authors indicated that phthalate exposure affects papillary thyroid cancer development (Miao et al. 2020). Taken together, although the summarized studies indicate a positive correlation between phthalates and thyroid cancer, the available data cannot reveal a definitive association between chemical exposure and disease development due to a lack of evidence (Marotta et al. 2020).

### In vitro* and *in silico* studies*

Other studies have investigated phthalate exposure and thyroid functions using meta-analysis and in vitro and in silico methods. In a meta-analysis study with 13 articles, Kim et al. (2019) demonstrated an inverse association between urinary MEHP and MEHHP concentrations and TT4, while urinary 5-oxo-MEHP metabolite concentration was positively associated with thyrotropin. It was concluded that exposure to DEHP metabolites could be significantly correlated with the function of the HPT axis (Kim et al. 2019).

In vitro analysis using monkey fibroblast–derived CV-1 cells, Shen et al. (2009) showed that DEHP, DBP, and MBP can bind to TH receptor as antagonists with IC50 of 13 µM, 2.7 µM, and greater than 100 µM, respectively (Shen et al. 2009). Using the same cell line (CV-1), Ibhazehiebo and Koibuchi (2011) analyzed the impacts of DEHP exposure on THR-mediated gene expression and found that the low dose of DEHP (10^–7^ M) can suppress 30% of THR-mediated transcription. It was suggested that DEHP is a non-competitive inhibitor of THR as no dose-dependent activity was observed (Ibhazehiebo and Koibuchi 2011). Using in vitro and in silico approaches, Kambia et al. (2021) compared the effects of DEHP and DEHT metabolites on THRs. The in vitro assay (rat pituitary tumor cell line GH3) showed that DEHT metabolite MEHT has an antagonistic effect at below cytotoxic doses (2–5 µg/mL), while MEHP was found to be partial agonist between 10 and 20 µg/mL. It was also demonstrated that 5-OH-MEHP primarily acts as an agonist at a concentration of 0.2 µg/mL, and at higher doses, it shows synergetic effect with T3. These in vitro data were also confirmed by in silico docking results (Kambia et al. 2021).

## Conclusion

TH regulates almost every part of the body, and it is implicated in a wide range of physiological functions. From the available data, it is evident that plasticizers negatively regulate the TH system. This is a matter of concern as the production of plasticizers is expected to increase and new alternatives are also introduced whose toxicity is not fully elucidated. Plasticizers could have a long-lasting impact on organisms by affecting their TH homeostasis. TH action can be regulated at different levels (Fig. 2); hence, plasticizers’ mechanisms of action to impact the TH system could be diverse. Plasticizers could demonstrate positive and/or negative association with serum levels of T3 and T4 and alter hormone levels, including TSH and TRH in a sex-specific manner. Plasticizers could also affect the genes and proteins involved in the TH system and cause thyroid follicular cell hypertrophy and hyperplasia. The available data also indicate age-dependent impacts of plasticizers on the TH system as several plasticizers were detected in urine and serum of children and adults at different levels. Since plasticizers affect the TH system, they can be classed under endocrine disruptors. Hence, a thorough analysis of old and new emerging plasticizers in the regulation of the thyroid system is required.

## Data Availability

Not applicable.
